# Clinicopathological Patterns and Outcomes in Patients with Lupus Nephritis and Hyperuricemia

**DOI:** 10.3390/jcm11113075

**Published:** 2022-05-30

**Authors:** Qiong Wen, Xueqing Tang, Qian Zhou, Wei Chen, Xueqing Yu

**Affiliations:** 1 Department of Nephrology, The First Affiliated Hospital, Sun Yat-sen University, Guangzhou 510080, China; wenqiong@mail.sysu.edu.cn (Q.W.); tangxueqing2010@aliyun.com (X.T.); yuxq@mail.sysu.edu.cn (X.Y.); 2 NHC Key Laboratory of Clinical Nephrology (Sun Yat-sen University), Guangzhou 510080, China; 3 Guangdong Provincial Key Laboratory of Nephrology, Guangzhou 510080, China; 4 Department of Nephrology, The First Affiliated Hospital of Shandong First Medical University (Shandong Provincial Qianfoshan Hospital), Jinan 250014, China; 5 Clinical Trials Unit, The First Affiliated Hospital, Sun Yat-sen University, Guangzhou 510080, China; zhouq49@mail.sysu.edu.cn

**Keywords:** lupus nephritis, hyperuricemia, uric acid, outcomes, risk factors

## Abstract

A limited number of large cohort studies have reported the clinicopathological characteristics and prognosis of patients with lupus nephritis (LN) and hyperuricemia (HUA). In this retrospective cohort study, 1297 LN patients were enrolled from January 1996 to December 2011 in the First Affiliated Hospital of Sun Yat-Sen University, and HUA occurred in 649 (50.04%) of these 1297 LN patients. Compared to patients without HUA, those with HUA presented with higher blood pressure and triglyceride levels, lower hemoglobin and serum albumin levels, worse renal function, more severe hematuria and proteinuria, higher lupus activity, and more positive antiphospholipid antibody. Pathologically, HUA cases presented more crescents, a higher degree of mesangial matrix, endothelial cell proliferation, and inflammatory cell infiltration. During the 52-month follow-up, the 5-year and 10-year incidence rates of renal endpoint events were 11.1% and 19.5% in the HUA group, and 8.3% and 13.8% in the non-HUA group, respectively (*p* = 0.073). In addition, the 5-year and 10-year mortality rates did not differ significantly between the HUA (12.0% and 18.2%) and non-HUA (12.2% and 17.5%) groups, respectively. This study verified that HUA was not an independent risk for poor clinical outcomes, and steroids that delay the deterioration of renal function did not affect the survival of these patients.

## 1. Introduction

Lupus nephritis (LN) is the most common form of serious organ damage caused by systemic lupus erythematosus (SLE), a systemic autoimmune inflammatory disease with a broad range of clinical presentations [1]. The frequency of renal involvement in SLE has been reported to be up to 50–60% [2], and approximately 20% of patients with LN develop end-stage renal disease (ESRD) that requires renal replacement therapy within 10 years of presentation [3]. Patients with ESRD have a 26-fold higher risk of death, which is twice that of malignancy or cardiovascular diseases [4].

Hyperuricemia (HUA) is a type of metabolic disease and an established cause of gout and urate nephropathy. HUA can also directly lead to a high risk of onset of hypertension and diabetes, and can contribute to renal injury [5]. Furthermore, uric acid (UA) levels are related to the prognosis of diseases, such as coronary heart disease [6], stroke [7], chronic kidney disease (CKD) [8], ESRD [9], and IgA nephropathy (IgAN) [10]. A previous study detected an HUA prevalence of 16.1% in patients with SLE, in whom HUA was significantly associated with stroke and peripheral neuropathy [11]. In addition, a high level of UA in SLE was also associated with pulmonary hypertension [12]. In patients with LN and CKD of stages 1–3, the HUA prevalence (40.1%) was higher than that in SLE in general, in which renal underexcretion HUA was the most prevalent subtype [13]. However, the association between LN and HUA remains inadequately studied. Liu et al. found that HUA in LN patients was associated with renal insufficiency, metabolic disorder, and lupus itself [13]. A study from Korea showed that every 1 mg/dL increase in the baseline UA level increased the progression risk of LN by 15.1% [14]. Additionally, a prospective cohort study by Elnady et al. found associations between high levels of serum UA and LN onset and new onset of renal damage [15].

It is well-known that UA is excreted mainly through the kidney, and HUA is perfectly correlated with a lower estimated glomerular filtration rate (eGFR). In addition to eGFR, it is still controversial whether HUA is independently associated with renal prognosis. Moreover, studies on the relationship between HUA and survival in patients with LN are scarce. Large sample studies on the prevalence of HUA in LN patients and detailed descriptions of the clinicopathological characteristics of LN with HUA are still lacking. In addition, the risk factors for clinical outcomes among patients with LN and HUA remain unclear. Thus, this study aimed to investigate the clinicopathological features and outcomes of patients with LN and HUA, and to explore the risk factors for mortality and renal failure in these patients.

## 2. Patients and Methods

In this single-center, observational cohort study, a total of 1297 patients (≥14 years old), who were diagnosed with SLE (defined by the 1997 revised American College of Rheumatology criteria) and LN according to the clinical manifestations, laboratory findings, and renal histopathological changes, were enrolled between 1 January 1996 and 31 December 2011 in the First Affiliated Hospital of Sun Yat-sen University. Patients with an age < 14 years, drug-induced SLE, previous renal transplantation, merged malignant tumors, or missing data such as serum UA levels were excluded.

The clinical and demographic data, such as age, gender, blood pressure (BP), and medications, were collected retrospectively at the time of renal biopsy. The laboratory data at baseline included 24-h urinary protein excretion and serum levels of creatinine, blood urea nitrogen (BUN), hemoglobin, cholesterol, triglycerides (TG), high-density lipoprotein-cholesterol (HDL-c), low-density lipoprotein-cholesterol (LDL-c), and UA. The serum levels of antinuclear antibody (ANA), anti-double-stranded DNA (anti-dsDNA) antibody, anti-extractable nuclear antigen antibody (the Smith (Sm)), and complement C3 were also recorded.

Patients were classified as hyperuricemic if the fasting serum UA level was >420 µmol/L (7 mg/dL) in men and postmenopausal women, or >360 µmol/L (6 mg/dL) in premenopausal women twice on different days. The Modification of Diet in Renal Disease (MDRD) formula was used to calculate the eGFR [16]. CKD was defined and staged based on the K/DOQI clinical practice guidelines [17]. The SLE disease activity index (SLEDAI) was employed based on the SLEDAI-2000 system [18]. Renal biopsy reports based on light, immunofluorescence, and electron microscopy findings were also obtained. The World Health Organization (WHO) revised criteria were used to classify the renal biopsy specimens [19]. LN was pathologically classified according to the 2003 International Society of Nephrology and the Renal Pathology Society (ISN/RPS) pathological classification system [20]. The composite primary outcome included renal endpoint events, defined as doubling of serum creatinine, eGFR <15 mL/min/1.73 m^2^, dialysis, renal transplantation, or death.

### Statistical Analysis

Data are presented as the mean ± standard deviation (normal distribution) or median with 25th and 75th percentiles (non-normal distribution). Differences between two groups were analyzed using the *t*-test and Mann–Whitney U-test. Differences in the qualitative results, expressed as frequency with percentage, were compared using the chi-squared test, Fisher’s exact test, or the Kruskal–Wallis test as appropriate. Kaplan–Meier analysis with log-rank tests, as well as univariable and multivariable Cox regression analyses, were used to evaluate the effect of HUA on LN prognosis. Univariable and multivariable Cox regression analyses were also used to analyze the relationships between clinicopathological indices and HUA manifestations. A multivariable Cox proportional hazards model was adjusted for variables that may affect LN progression based on the references or clinical recognition and variables that were significant in univariable regression. Collinearity was analyzed to assess the correlations of variables with the other independent variables. Statistical analysis was performed using SPSS (version 16.0, SPSS Inc., Chicago, IL, USA), and statistical significance was expressed at *p*-value < 0.05.

## 3. Results

### 3.1. Clinical and Pathological Characteristics of LN Patients with and without HUA

From January 1996 to December 2011, a total of 1297 LN patients were enrolled in this study, and 649 of these patients presented with HUA. One patient had gout, and seven patients had kidney stones in the HUA group, and twelve patients had kidney stones in the non-HUA group. However, the components of kidney stones were not clear. Compared to LN patients without HUA, LN patients with HUA presented with higher blood pressure and triglyceride levels, lower hemoglobin and serum albumin levels, worse renal function, worse hematuria and proteinuria, higher lupus activity, and more positive antiphospholipid antibody (Table 1). Nearly 24% (311/1297) of the patients in our study had used immunosuppressants prior to renal biopsy. Moreover, 16.4% (213/1297) of patients with relapses before renal biopsy were also included in the study.

Significant differences were found between the two groups in the pathological findings (Table 2). The HUA group presented with more crescents, increased mesangial matrix, greater endothelial cell proliferation, and greater inflammatory cell infiltration compared with the non-HUA group, but no difference was found in the LN pathological grade between the two groups (*p* = 0.163).

### 3.2. Comparison of the Clinical Outcomes between LN Patients with and without HUA

For the analysis of clinical outcomes, 121 patients with ESRD based on eGFR <15 mL/min/1.73 m^2^, the need for dialysis, or renal transplantation at the time of renal biopsy were excluded. The clinical outcomes of the remaining 1176 LN patients were assessed on follow-up via telephone interviews or reviews of medical records. Finally, 820 patients were successfully followed up in this study.

**Figure 1:**
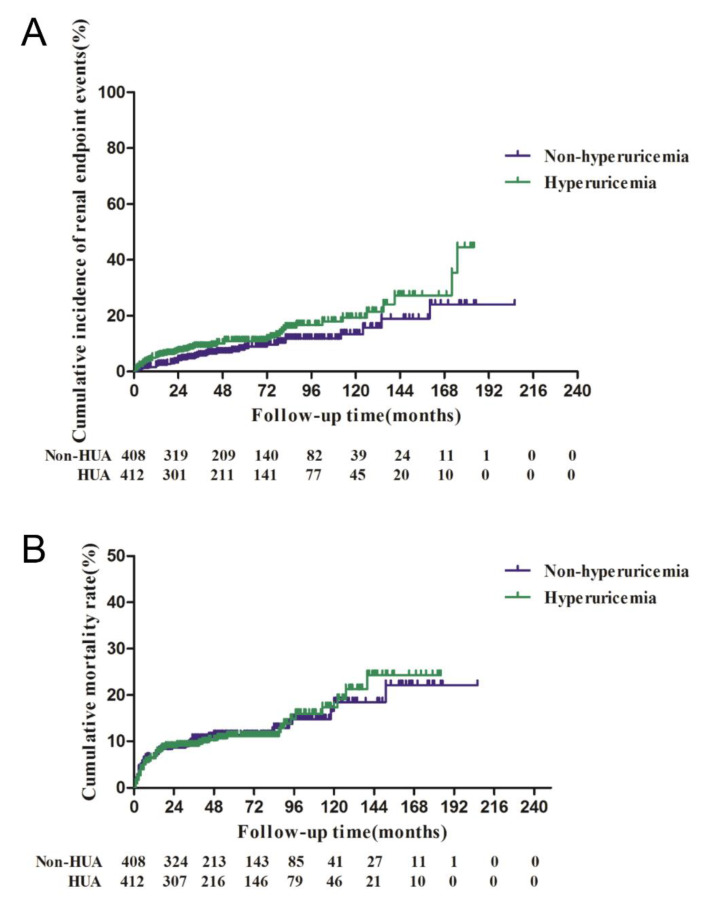
Kaplan–Meier survival functions of LN (lupus nephritis) patients with hyperuricemia (HUA) and without hyperuricemia. (**A**) Log-rank significance for renal endpoint events = 0.073; (**B**) Log-rank significance for death = 0.955.

During the 52-month (interquartile range (IQR), 28−87 months) follow-up for 412 cases with HUA (50.2%) and 408 cases without HUA (49.8%), the 5-year and 10-year incidence rates of renal endpoint events were 11.1% and 19.5% in the HUA group compared with 8.3% and 13.8% in the non-HUA group, respectively, with no significant difference between the two groups (*p* = 0.073; Figure 1A). In addition, the 5-year and 10-year mortality rates did not differ significantly (*p* = 0.955) between the HUA group (12.0% and 18.2%) and non-HUA group (12.2% and 17.5%; Figure 1B). Furthermore, relapse occurred in 42 patients in the HUA group and 50 patients in the non-HUA group during follow-up, with no statistically significant difference between the two groups (*p* = 0.383). Furthermore, using multivariate regression, we found that pathologic type Ⅳ (OR = 1.936; 95% CI, 1.061−3.533; *p* = 0.031) and Ⅴ + Ⅳ (OR = 3.624; 95% CI, 1.592−8.251; *p* = 0.002) were independent risk factors for recurrence in patients with LN during follow-up, but HUA had no effect on relapse (*p* = 0.365).

Cox regression analysis showed that HUA was not a risk factor for a renal endpoint event or death with or without adjustment for age, gender, disease course, BP, albumin, urinary protein, hemoglobin, LDL-c, eGFR (mL/min/1.73 m^2^), SLEDAI, and pathological classification (Table 3).

### 3.3. Risk Factors for Clinical Outcomes in Patients with LN and HUA

As previously mentioned, the 5-year and 10-year incidence rates of renal endpoint events were 11.1% and 19.5% in patients with HUA (Figure 1A). Cox multivariate regression analysis found that high eGFR (risk ratio (RR) = 0.984; 95% confidence interval (CI), 0.970−0.998; *p* = 0.025) and use of steroids (RR = 0.074; 95% CI, 0.014−0.387; *p* = 0.002) were independent protective factors against progression to renal endpoints in these patients (Table 4). Furthermore, the 5-year and 10-year mortality rates were 12.0% and 18.2% in the HUA group, and Cox regression analysis showed that only high eGFR (RR = 0.987; 95% CI, 0.977−0.998; *p* = 0.022) was an independent protective factor against death in patients with HUA (Table 4).

## 4. Discussion

In this study, we investigated the clinicopathological characteristics of LN patients with HUA, and explored the risk factors for mortality and the development of renal dysfunction in these patients. Among the 1297 patients with LN, approximately half of the patients had HUA. In comparison, the prevalence of HUA was 40.11% in a study by Liu et al. [13], and 37.3% in a study by Oh et al. [14]; these rates were lower than that in our study. As already known, UA is excreted predominantly by the kidneys, and its level increases gradually with a decrease in renal function. The difference in HUA prevalence in our study occurred mainly because we included LN patients with any stage of renal function (18.1% patients had CKD stages 4–5), whereas the two previous studies only included patients with normal kidney function or mild renal impairment (stages 1–3 of CKD). Furthermore, the prevalence of HUA in the CKD population or in the general population is much lower. In a community-based population study, the HUA prevalence in patients with stages 1–3 of CKD was 23.3% [21]. Data from the US National Health and Nutrition Examination Survey 2007–2008 showed that the prevalence of HUA in adults living in the US was over 21% [22]. The prevalence of HUA in Mainland China was 13.3% (95% CI: 11.9–14.6%) from 2000 to 2014 [23]. These results reveal that the cause of HUA in LN might not only be related to renal insufficiency, but also to lupus itself, geographical region, and economic level.

Compared to LN patients without HUA, we found that LN patients with HUA presented with higher BP and triglyceride levels; lower hemoglobin and serum albumin levels; worse renal function, hematuria, and proteinuria; higher activity of lupus; and more positive antiphospholipid antibody. A study from Korea showed similar results; their HUA group showed higher creatinine level and blood pressure, lower hemoglobin and serum albumin levels, a lower C3 level, higher anti-dsDNA antibody, lower eGFR, higher total cholesterol, and higher urine protein to creatinine ratio [14]. Liu et al. also reported more hypertension with increases in urine sediment, triglycerides, blood glucose, BUN, serum creatinine, phosphorus, parathyroid hormone, 24-h urinary albumin, 24-h urinary α1-microglobulin, and activity of urine *N*-acetyl-β*D*-glucosaminidase (NAG), along with lower eGFR, serum albumin, complement 3, 24-h urinary calcium, urinary volume, and urinary pH in patients with LN and HUA compared to those without HUA [13]. No significant difference was found in the sex ratio or age between LN patients with and without HUA in these two studies and our study. Differences in sample sizes, patient inclusion criteria, and variables included in baseline data also led to some differences among the studies.

This is the first study to show that more LN patients with HUA test positive for antiphospholipid antibodies. Although both HUA and antiphospholipid antibodies have been previously reported as risk factors for early atherosclerosis [24], the relationship between antiphospholipid antibodies and HUA was not well understood. In 2019, new SLE classification criteria published by the European League Against Rheumatism (EULAR) and ACR consisted of 22 diagnostic criteria with different weights, and included antiphospholipid antibodies in the diagnosis [25]. These classification criteria indicate that as an autoimmune antibody, the antiphospholipid antibody plays a critical role in lupus pathogenesis. Antiphospholipid antibodies, including anticardiolipin antibodies, lupus anticoagulant, and anti-beta-2 glycoprotein I antibodies, are directed against phospholipid-protein complexes. They can activate endothelial cells, monocytes, and platelets that lead to proinflammatory and prothrombotic phenotypes, and complement activation, which results in thrombosis [26]. Many biological actions respond to urate, including antioxidant effects, and immune and proinflammatory effects. Urate can be released from dying cells and it can aid the immune response and facilitate the recognition of apoptotic cells, which then ultimately form and deposit autoantibodies in the kidney [27]. In addition, HUA leads to the activation of the renin-angiotensin system (RAS) and down-regulation of nitric oxide (NO), which are responsible for blood vessel relaxation, induction of vascular smooth muscle cell proliferation, and endothelial cell dysfunction [28]. Therefore, HUA and antiphospholipid antibodies might play a role in the development of LN through both proinflammatory effects and endothelial cell injury.

Our study also found that the HUA group presented with increased proliferation of mesangial matrix and endothelial cells, and greater infiltration of inflammatory cells compared to the non-HUA group, but there was no difference in the LN pathological grade between the two groups. A retrospective study also showed that LN patients with HUA had significantly higher renal pathological scores based on the 2003 ISN/RPS pathological classification system, including active index, chronic index, and tubulointerstitial lesions [29]. Another study from China reported different results [13]. The numbers of patients with pathological classes (I, II, III, IV, V, (II + V), (III + V), and (IV + V)) who had HUA were 1, 0, 7, 19, 5, 0, 12, and 9, respectively, whereas the numbers of patients with classes (I, II, III, IV, V, (II + V), (III + V), and (IV + V)), who did not have HUA were 1, 9, 10, 18, 19, 3, 20, and 8, respectively. A statistical difference was found in the constituent ratio of pathological types between the two groups. Instead, no significant difference was found between the two groups in the other pathological parameters, such as crescents, mesangial proliferation, endothelial proliferation, leukocyte infiltration, and small vascular lesions. However, both global sclerosis and tubular interstitial lesions showed no significant difference in that study and our study. In addition to the differences in sample size and inclusion criteria, one reason for these differences could be that the previous study used continuous variables, whereas we used categorical variables.

HUA is known to be associated with a poor prognosis. Shimizu et al. demonstrated that HUA can independently predict all-cause mortality (hazard ratio = 1.98, *p* = 0.039) in patients with heart failure, and preserved ejection fraction [30]. In a cohort study, Rodenbach et al. reported that HUA as a previously undescribed independent risk factor for faster progression of CKD in children and adolescents. Compared to the participants with initial UA levels <5.5 mg/dL, those with UA levels >5.5 mg/dL had 17% and 38% shorter times to a >30% decrease in eGFR and initiation of renal replacement therapy, respectively [31]. Nevertheless, few studies have examined the clinical outcomes of LN patients with HUA. In the present study, the 5-year and 10-year incidence rates of renal endpoint events were 11.1% and 19.5% in the HUA group versus 8.3% and 13.8% in the non-HUA group, respectively, and the 5-year and 10-year mortality rates were 12.0% and 18.2% in the HUA group versus 12.2% and 17.5% in the non-HUA group, respectively. No significant differences were found between the two groups. Finally, multivariate Cox regression analysis showed that HUA was not a risk factor for renal endpoint events or death. This result may be because the most common cause of renal endpoint, as events or death among LN patients were renal injury, lupus itself, and infection, as well as the short-term follow-up. However, a study from Korea indicated that the risk of LN progression (defined as the initiation of dialysis or kidney transplantation) was increased by nearly 15.1% by high serum UA [14]. The above-described study also found that the serum UA level was an independent risk factor for LN progression in women, but not in men [14]. We had also attempted stratified analysis by age, sex, and renal function, but we failed to identify an independent correlation between HUA and prognosis. A recent study from a small sample also detected that the serum UA level <6.05 mg/dL at 12 months can predict a positive long-term renal outcome (creatinine clearance ≥90.0 mL/min/1.73 m^2^ within 7 years) in LN patients [32]. The reasons for the different results were that these two studies had different definitions of the primary endpoint, and used specific values of UA in Cox regression analysis, whereas our study directly used HUA as a variable in the regression. Moreover, it is unclear whether patients with LN and HUA should be treated with urate-lowering therapy (ULT), as the benefits of ULT on prognosis are still not clear. A review of randomized clinical trials revealed that treatment with ULT conferred consistent clinical benefits. Therefore, routine measurement of serum urate levels is recommended for patients with CKD who consider starting ULT [33]. Thus, we could consider evaluating the benefit of ULT in patients with LN and HUA in the future.

No previous studies on the risk factors for renal outcome and death in LN patients with HUA could be found currently. In the present study, we revealed that low eGFR and no use of steroids could independently predict the progression to renal endpoint events in patients with HUA, and low eGFR was the only independent risk factor for death in these patients.

This study has several limitations. First, we used a retrospective single-center design; thus, our findings cannot readily be applied to a wider population. We could also not prove causality between HUA and LN progression; the inability to prove causality is a trait of all observational studies. Second, the follow-up time was short, and some differences could not be fully assessed. For example, a significant difference in the incidence of renal endpoint events was not detected between the HUA and non-HUA groups. Third, the intake of some foods and ULT that can influence serum UA levels was not considered, and thus, we did not analyze whether lowering UA improves the outcomes. Therefore, a prospective cohort study with a long follow-up is needed.

## 5. Conclusions

In this study, half of the patients with LN had HUA; however, HUA was not an independent risk for clinical outcomes. Low eGFR was able to independently predict poor outcomes for patients with LN and HUA. Further, steroid use did not affect patient survival, although it could partially delay the deterioration of renal function.

## Figures and Tables

**Table 1:** Clinical characteristics of LN patients in the hyperuricemia and non-hyperuricemia groups.

	All (*n* = 1297)	Hyperuricemia (*n* = 649, 50.04%)	Non-Hyperuricemia (*n* = 648, 49.96%)	*p*
Age (years)	29.0 (22.0, 39.0)	27.0 (20.0, 37.0)	28.0 (22.0, 38.0)	0.525
Male (*n*, %)	219, 16.9%	114, 17.6%	105, 16.2%	0.368
Disease course (months)	4 (1, 24)	4 (1, 24)	4 (1, 24)	0.616
Systolic BP (mmHg)	128.0 (114.0, 143.0)	130.0 (115.0, 145.0)	120.0 (110.0,138.3)	<0.001
Diastolic BP (mmHg)	80.0 (70.0, 91.0)	83.0 (75.0, 94.0)	80.0 (70.0,90.0)	<0.001
Hemoglobin (g/L)	95.0 (78.0, 114.0)	93.0 (76.3, 108.0)	108.5 (93.0, 112.0)	<0.001
Serum albumin (g/L)	27.0 (22.0. 33.0)	25.0 (20.0, 31.0)	29.9 (24.0,35.0)	<0.001
Cholesterol (mmol/L)	5.6 (4.4, 7.3)	5.9 (4.6, 7.5)	5.7 (4.2, 7.3)	0.635
Triglycerides (mmol/L)	2.1 (1.4, 3.1)	2.2 (1.5, 3.5)	1.9 (1.3, 2.6)	<0.001
SCr (µmol/L)	94.0 (65.0, 178.0)	106.0 (74.3,182.3)	72.0 (56.0, 107.5)	<0.001
eGFR (mL/min. 1.73 m^2^)	93.8 (42.2, 126.0)	68.0 (32.4,115.4)	116.0 (74.9, 135.5)	<0.001
CKD staging (*n*, %)				
Stage 1	663, 51.1%	257, 39.6%	406, 62.8%	<0.001
Stage 2	186, 14.3%	99, 15.3%	87, 13.4%	
Stage 3	210, 16.2%	140, 21.6%	70, 10.8%	
Stage 4	107, 8.2%	75, 11.6%	32, 4.9%	
Stage 5	129, 9.9%	77, 11.9%	52, 8.0%	
Urine RBC >3+ (*n*, %)	259, 20.0%	148, 22.8%	111,17.1%	0.004
Urinary protein (g/24h)	1.67 (0.7, 3.3)	2.0 (1.0, 3.8)	1.3 (0.5, 2.8)	0.001
SLEDAI score	14.0 (10.0, 18.0)	16.0 (12.0,19.0)	14.0 (10.0, 18.0)	0.002
dsDNA (positive) (*n*, %)	958, 73.9%	514, 79.2%	444, 68.5%	0.001
DNP (positive) (*n*, %)	237, 18.3%	122, 18.8%	115, 17.7%	0.601
SSA (positive) (*n*, %)	543, 41.9%	269, 41.4%	274, 42.3%	0.542
SSB (positive) (*n*, %)	238, 18.4%	120, 18.5%	118, 18.2%	0.841
SM (positive) (*n*, %)	299, 23.1%	147, 22.7%	152, 23.5%	0.941
RNP (positive) (*n*, %)	382, 29.5%	191, 29.4%	191, 29.5%	0.935
C3 (g/L)	0.5 (0.3, 0.9)	0.4 (0.3, 0.6)	0.5 (0.3, 0.7)	0.035
ACL-IgM (positive) (*n*, %)	194, 15.0%	119, 18.3%	35, 12.8%	0.036
ACL-IgG (positive) (*n*, %)	258, 19.9%	156, 24.0%	75, 11.6%	0.011
P-ANCA(MPO) (positive) (*n*, %)	75, 5.8%	40, 6.2%	35, 5.4%	0.849
C-ANCA(PR3) (positive) (*n*, %)	31, 2.4%	21, 3.2%	10, 1.5%	0.663
Steroids (*n*, %)	1255, 96.8%	625, 96.3%	630, 97.2%	0.434
Immunosuppressants (*n*, %)	723, 55.7%	369. 56.9%	354, 54.6%	0.356
Cyclophosphamide	433, 33.4%	219, 33.7%	214, 33.0%	0.961
Mycophenolate	171, 13.2%	86, 13.3%	85, 13.1%	
Cyclosporine	26, 20.0%	14, 2.2%	12, 1.9%	
Tacrolimus	15, 1.2%	9, 1.4%	6, 0.9%	
Others	78, 6.0%	41, 6.3%	37, 5.7%	
ACEI/ARB (*n*, %)	654, 50.4%	353, 54.4%	301, 46.5%	0.035

BP = blood pressure; RBC = red blood cell; SCr = serum creatinine; ACEI = angiotensin-converting enzyme inhibitor; ARB = angiotensin receptor blocker. a eGFR (mL/min/1.73 m^2^) = 175 × (plasma creatinine) − 1.234 × age − 0.179 × 0.79 (if female). *p*-values < 0.05 were considered statistically significant.

**Table 2:** Pathological characteristics of LN (lupus nephritis) patients in the hyperuricemia and non-hyperuricemia groups.

	All (*n* = 1297)	Hyperuricemia (*n* = 649, 50.04%)	Non-Gyperuricemia (*n* = 648, 49.96%)	*p*
Global glomerular sclerosis (%)	0 (0, 11.1)	0 (0. 14.3)	0 (0, 27.3)	0.082
Crescent (%)	3.5 (0. 18.1)	0 (0, 23.3)	0 (0, 18.8)	<0.001
Mesangial cell and matrix proliferation (*n*, %)				
<25%	357, 42.9%	145, 33.5%	212, 53.1%	<0.001
25–50%	277, 33.3%	155, 35.8%	122, 30.6%	
≥50%	198, 23.8%	133, 30.7%	65, 16.3%	
Endothelial cell proliferation (*n*, %)				
<25%	226, 27.2%	89, 20.6%	137, 34.3%	<0.001
25–50%	366, 44.0%	183, 42.3%	183, 45.9%	
≥50%	240, 28.8%	161, 37.2%	79, 19.8%	
Leukocyte infiltration (*n*, %)				
none	287, 34.5%	132, 30.7%	155, 38.8%	<0.001
<25%	339, 40.7%	166, 38.3%	173, 43.4%	
25–50%	176, 21.2%	110, 25.4%	66, 16.5%	
≥50%	30, 3.6%	25, 5.8%	5, 1.3%	
Interstitial fibrosis (*n*, %)				
0	200, 24.0%	160, 37.0%	40, 10.0%	0.052
<25%	476, 57.2%	192, 44.3%	284, 71.2%	
25–50%	102, 12.3%	57, 13.2%	45, 11.3%	
50–75%	34, 4.1%	16, 3.7%	18, 4.5%	
≥75%	19, 2.3%	7, 1.6%	12, 3.0%	
Tubular atrophy (*n*, %)				
0	330, 39.7%	163, 37.6%	167, 41.9%	0.120
<25%	376, 45.2%	183, 42.3%	193. 48.4%	
25-50%	89, 10.7%	65, 15.0%	24, 6.0%	
50–75%	26, 3.1%	15, 3.5%	11, 2.8%	
≥75%	11, 1.3%	7, 1.6%	4, 1.0%	
Interstitial infiltrates (*n*, %)				
0	200, 24.0%	85, 19.6%	115, 28.8%	<0.001
<25%	476, 57.2%	243, 56.1%	233, 58.4%	
25–50%	102, 12.3%	74, 17.1%	28, 7.0%	
50–75%	34, 4.1%	20, 4.6%	14, 3.5%	
≥75%	19, 2.3%	11, 2.5%	8, 2.0%	
Pathological grade				
Ⅰ	5, 0.9%	1, 0.4%	4, 1.0%	0.163
Ⅱ	55, 10.3%	17, 6.2%	38, 9.5%	
Ⅲ	51, 9.6%	18, 6.5%	33, 8.3%	
Ⅳ	227, 42.6%	144, 52.4%	83, 20.8%	
Ⅴ	73, 13.7%	27, 9.8%	46, 11.5%	
Ⅵ	10, 1.9%	7, 2.5%	3, 0.8%	
Ⅴ + Ⅲ	47, 8.8%	21, 7.6%	26, 6.5%	
Ⅴ + Ⅳ	65, 12.2%	40, 14.5%	25, 6.3%	

**Table 3:** Hyperuricemia as a risk factor for clinical outcomes, as assessed by Cox regression models.

	Unadjusted		Model 1		Model 2	
	HR (95%CI)	*p*	HR (95%CI)	*p*	HR (95%CI)	*p*
Death						
hyperuricemia	1.01 (0.69, 1.48)	0.955	1.06 (0.72, 1.55)	0.763	0.85 (0.52, 1.39)	0.517
Renal endpoint event						
hyperuricemia	1.46 (0.96, 2.23)	0.075	1.49 (0.98, 2.27)	0.062	1.35 (0.80, 2.28)	0.255

Model 1: adjusted for age, gender, and course of disease. Model 2: adjusted for age, gender, course of disease, BP, albumin, urinary protein, hemoglobin, LDL-c, eGFR (mL/min/1.73 m^2^), SLEDAI, and pathological classification. HR, hazard ratio; CI, confidence interval.

**Table 4:** Factors influencing clinical outcomes among LN(lupus nephritis) patients with hyperuricemia.

(a) Cox Regression for Renal Endpoint Event			
Parameter	Multivariable Analysis		
	RR	95% CI	*p*
Age (years)	1.011	0.367–2.788	0.983
Male	1.015	0.987–1.044	0.298
Disease course (months)	1.000	0.983–1.004	0.946
HBP	1.462	0.666–3.209	0.344
Urinary protein (g/24 h)	1.064	0.918–1.235	0.409
Hemoglobin (g/L)	0.991	0.968–1.014	0.423
LDL-c (mmol/L)	1.077	0.829–1.400	0.579
eGFR (mL/min. 1.73 m^2^)	0.984	0.970–0.998	0.025
SLEDAI score	0.992	0.911–1.079	0.844
Pathological grade	0.998	0.995–1.001	0.285
Global glomerular sclerosis %	2.089	0.218–20.06	0.523
Tubular atrophy %	1.573	0.909–2.724	0.106
Steroids	0.074	0.014–0.387	0.002
**(b) Cox Regression for Death**			
**Parameter**	**Multivariable Analysis**		
	**RR**	**95% CI**	***p***
Male	1.214	0.515–2.858	0.658
Age (years)	1.022	0.994–1.050	0.133
Disease course (months)	0.999	0.989–1.010	0.895
Urinary protein (g/24 h)	0.966	0.846–1.102	0.605
eGFR (mL/min. 1.73 m^2^)	0.987	0.977–0.998	0.022
Tubular atrophy %	0.863	0.515–1.447	0.576

RR, relative risk; HBP, high blood pressure; LDL-c, low-density lipoprotein-cholesterol; eGFR, estimated glomerular filtration rate; CI, confidence interval.

## Data Availability

The datasets generated and analyzed during the current study are not publicly available as none of the data types require uploading to a public repository but are available from the corresponding author on reasonable request.

## References

[1] Rahman A., Isenberg D.A. (2008). Systemic lupus erythematosus. N. Engl. J. Med..

[2] Almaani S., Meara A., Rovin B.H. (2017). Update on Lupus Nephritis. Clin. J. Am. Soc. Nephrol..

[3] Clark W.F., Sontrop J.M. (2008). What have we learned about optimal induction therapy for lupus nephritis (III through V) from randomized, controlled trials?. Clin. J. Am. Soc. Nephrol..

[4] Burgos P.I., McGwin G., Pons-Estel G.J., Reveille J.D., Alarcón G.S., Vilá L.M. (2011). US patients of Hispanic and African ancestry develop lupus nephritis early in the disease course: Data from LUMINA, a multiethnic US cohort (LUMINA LXXIV). Ann. Rheum. Dis..

[5] Abeles A.M. (2015). Hyperuricemia, gout, and cardiovascular disease: An update. Curr. Rheumatol. Rep..

[6] Kim S.Y., Guevara J.P., Kim K.M., Choi H.K., Heitjan D.F., Albert D.A. (2010). Hyperuricemia and coronary heart disease: A systematic review and meta-analysis. Arthritis Care Res..

[7] Li M., Hou W., Zhang X., Hu L., Tang Z. (2014). Hyperuricemia and risk of stroke: A systematic review and meta-analysis of prospective studies. Atherosclerosis.

[8] Srivastava A., Kaze A.D., McMullan C.J., Isakova T., Waikar S.S. (2018). Uric Acid and the Risks of Kidney Failure and Death in Individuals With CKD. Am. J. Kidney Dis..

[9] Jeong H.Y., Cho H.J., Kim S.H., Kim J.C., Lee M.J., Yang D.H., Lee S.Y. (2017). Association of serum uric acid level with coronary artery stenosis severity in Korean end-stage renal disease patients. Kidney Res. Clin. Pract..

[10] Russo E., Drovandi S., Salvidio G., Verzola D., Esposito P., Garibotto G., Viazzi F. (2020). Increased serum uric acid levels are associated to renal arteriolopathy and predict poor outcome in IgA nephropathy. Nutr. Metab. Cardiovasc. Dis..

[11] Sheikh M., Movassaghi S., Khaledi M., Moghaddassi M. (2015). Hyperuricemia in systemic lupus erythematosus: Is it associated with the neuropsychiatric manifestations of the disease?. Rev. Bras. Reumatol..

[12] Kim K.J., Baek I.W., Park Y.J., Yoon C.H., Kim W.U., Cho C.S. (2015). High levels of uric acid in systemic lupus erythematosus is associated with pulmonary hypertension. Int. J. Rheum. Dis..

[13] Liu S., Gong Y., Ren H., Zhang W., Chen X., Zhou T., Li X., Chen N. (2017). The prevalence, subtypes and associated factors of hyperuricemia in lupus nephritis patients at chronic kidney disease stages 1–3. Oncotarget.

[14] Oh T.R., Choi H.S., Kim C.S., Ryu D.R., Park S.H., Ahn S.Y., Kim S.W., Bae E.H., Ma S.K. (2020). Serum Uric Acid is Associated with Renal Prognosis of Lupus Nephritis in Women but not in Men. J. Clin. Med..

[15] Elnady B., Almalki A., Abdel-Fattah M.M., Desouky D.E., Attar M. (2021). Serum uric acid as a sensitive concordant marker with lupus nephritis and new onset of renal damage: A prospective cohort study. Clin. Rheumatol..

[16] National Kidney Foundation (2002). K/DOQI clinical practice guidelines for chronic kidney disease: Evaluation, classification, and stratification. Am. J. Kidney Dis..

[17] KDIGO Working Group (2012). Section 2: AKI Definition. Kidney Int. Suppl..

[18] Gladman D.D., Ibañez D., Urowitz M.B. (2002). Systemic lupus erythematosus disease activity index 2000. J. Rheumatol..

[19] Churg J., Bernstein J., Glassock R. (1995). Renal Disease: Classification and Atlas of Glomerular Disease.

[20] Weening J.J., D’Agati V.D., Schwartz M.M., Seshan S.V., Alpers C.E., Appel G.B., Balow J.E., Bruijn J.A.N.A., Cook T., Ferrario F. (2004). The classification of glomerulonephritis in systemic lupus erythematosus revisited. J. Am. Soc. Nephrol..

[21] Tsumuraya Y., Hirayama T., Tozuka E., Furuta W., Utsugi S., Tsuchiya A., Hishida A., Kumagai H. (2015). Impact of hyperuricaemia on the chronic kidney disease-associated risk factors in a community-based population. Nephrology.

[22] Zhu Y., Pandya B.J., Choi H.K. (2011). Prevalence of gout and hyperuricemia in the US general population: The National Health and Nutrition Examination Survey 2007–2008. Arthritis Rheum..

[23] Liu R., Han C., Wu D., Xia X., Gu J., Guan H., Shan Z., Teng W. (2015). Prevalence of Hyperuricemia and Gout in Mainland China from 2000 to 2014: A Systematic Review and Meta-Analysis. BioMed Res. Int..

[24] Serikova S., Kozlovskaia N.L., Shilov E.M. (2008). Lupus nephritis as a factor of atherosclerosis risk in patients with systemic lupus erythematosus. Ter. Arkhiv.

[25] Aringer M., Costenbader K., Daikh D., Brinks R., Mosca M., Ramsey-Goldman R., Smolen J.S., Wofsy D., Boumpas D.T., Kamen D.L. (2019). 2019 European League Against Rheumatism/American College of Rheumatology Classification Criteria for Systemic Lupus Erythematosus. Arthritis Rheumatol..

[26] Sammaritano L.R. (2020). Antiphospholipid syndrome. Best Pract. Res. Clin. Rheumatol..

[27] Johnson R.J., Bakris G.L., Borghi C., Chonchol M.B., Feldman D., Lanaspa M.A., Merriman T.R., Moe O.W., Mount D.B., Lozada L.G.S. (2018). Hyperuricemia, Acute and Chronic Kidney Disease, Hypertension, and Cardiovascular Disease: Report of a Scientific Workshop Organized by the National Kidney Foundation. Am. J. Kidney Dis..

[28] Dos Santos M., Veronese F.V., Moresco R.N. (2020). Uric acid and kidney damage in systemic lupus erythematosus. Clin. Chim. Acta.

[29] Xie T., Chen M., Tang X., Yin H., Wang X., Li G., Li J., Zuo X., Zhang W. (2016). Hyperuricemia is an independent risk factor for renal pathological damage and poor prognosis in lupus nephritis patients. Zhong Nan Da Xue Xue Bao Yi Xue Ban.

[30] Shimizu T., Yoshihisa A., Kanno Y., Takiguchi M., Sato A., Miura S., Nakamura Y., Yamauchi H., Owada T., Abe S. (2015). Relationship of hyperuricemia with mortality in heart failure patients with preserved ejection fraction. Am. J. Physiol. Heart Circ. Physiol..

[31] Rodenbach K.E., Schneider M.F., Furth S.L., Moxey-Mims M.M., Mitsnefes M.M., Weaver D.J., Warady B.A., Schwartz G.J. (2015). Hyperuricemia and Progression of CKD in Children and Adolescents: The Chronic Kidney Disease in Children (CKiD) Cohort Study. Am. J. Kidney Dis..

[32] Ugolini-Lopes M.R., Gavinier S.S., Leon E., Viana V.T., Borba E.F., Bonfá E. (2019). Is serum uric acid a predictor of long-term renal outcome in lupus nephritis?. Clin. Rheumatol..

[33] Sato Y., Feig D.I., Stack A.G., Kang D.H., Lanaspa M.A., Ejaz A.A., Sánchez-Lozada L.G., Kuwabara M., Borghi C., Johnson R.J. (2019). The case for uric acid-lowering treatment in patients with hyperuricaemia and CKD. Nat. Rev. Nephrol..

